# Draft Genome Sequences of Lactobacillus delbrueckii subsp. *bulgaricus* Strains CBC-LB69 and CBC-LB8, Isolated from Homemade Dairy Foods in Bulgaria

**DOI:** 10.1128/MRA.00876-20

**Published:** 2020-11-05

**Authors:** Hanan R. Shehata, Richmond A. Chandler, Steven G. Newmaster

**Affiliations:** a NHP Research Alliance, College of Biological Sciences, University of Guelph, Guelph, Ontario, Canada; b Department of Microbiology, Faculty of Pharmacy, Mansoura University, Mansoura, Egypt; c Chandler Biopharmaceutical Corporation, Milton, Ontario, Canada; University of Maryland School of Medicine

## Abstract

Here, we report the draft genome sequences of Lactobacillus delbrueckii subsp. *bulgaricus* strains CBC-LB69 and CBC-LB8. The strains were isolated from naturally processed, homemade dairy foods in Bulgaria. The two genome assemblies each resulted in 39 contigs with total lengths of 1,752,493 and 1,759,908 bp and GC contents of 49.80% and 49.90%, respectively.

## Abstract (precis)

Here, we report the ndraft genome sequences of Lactobacillus delbrueckii subsp. *bulgaricus* strains CBC-LB69 and CBC-LB8. The strains were isolated from naturally processed, homemade dairy foods in Bulgaria. The two genome assemblies each resulted in 39 contigs, with total lengths of 1,752,493 and 1,759,908 bp and GC contents of 49.80% and 49.90%, respectively.

## ANNOUNCEMENT

Lactobacillus delbrueckii subsp. *bulgaricus* is a common lactic acid bacterium widely used as a starter culture in yogurt and dairy products (1, 2) and was reported to have health benefits such as anti-Helicobacter pylori activity (3), immunity enhancement (4–6), and reduction of allergies to dairy products (7).

Strains CBC-LB69 and CBC-LB8 were isolated from naturally processed, homemade dairy foods from ecologically clean preserves in industrial pollution-free and chemically untampered areas. Strain CBC-LB69 was isolated from Pirin Mountain, Bulgaria, in September 1969, and strain CBC-LB8 was isolated from Strandja Mountain, Bulgaria, in April 1980. Both strains were isolated on MRS agar plates incubated anaerobically at 44°C for 72 h (8) and were found to survive and colonize the human intestinal tract (9).

In this paper, we present the genome sequences of Lactobacillus delbrueckii subsp. *bulgaricus* strains CBC-LB69 and CBC-LB8. The genomes were sequenced to better understand the genetic basis of their probiotic health effects and their safety for human consumption.

Both strains were obtained from Chandler Biopharmaceutical Corporation as lyophilized powder. Genomic DNA was extracted from 50 mg of lyophilized powder using a NucleoSpin food kit (740945.50; Macherey-Nagel, Germany). The DNA was quantified using a Qubit 4.0 fluorometer and submitted to the Advanced Analysis Centre, University of Guelph (Guelph, ON, Canada), for library preparation and sequencing on the Illumina MiSeq platform using an Illumina Nextera XT kit and Illumina MiSeq v3 600-cycle reagent kit (2 × 300-bp reads).

The sequencing data were analyzed using CLC Genomics Workbench v20.0.3 (Qiagen Bioinformatics). Default parameters were used except where otherwise noted. The reads were quality trimmed to remove the low-quality sequences (limit = 0.05 base-calling error probability), allowing a maximum of 2 ambiguous nucleotides. The total number of paired-end reads from strains CBC-LB69 and CBC-LB8 were 2,308,638 and 2,134,850 before quality trimming and 2,279,307 and 2,122,703 after quality trimming, respectively. High-quality reads with totals of 270,907,719 and 322,302,876 bases were assembled using CLC *de novo* assembly with default parameters, achieving >150× coverage. Each genome assembly resulted in 39 contigs. The CBC-LB69 draft genome sequence has a total length of 1,752,493 bp, a GC content of 49.80%, and an *N*_50_ value of 93,636 bp. The CBC-LB8 draft genome sequence has a total length of 1,759,908 bp, a GC content of 49.90%, and an *N*_50_ value of 87,463 bp.

Full-length 16S rRNA gene sequences were extracted from the genome sequences using the ContEst16S tool (10) and were BLAST searched (using MegaBLAST against the nucleotide collection database and the rRNA/ITS databases) on GenBank to confirm the species identity as Lactobacillus delbrueckii subsp. *bulgaricus* (11). The genomes were annotated using the NCBI Prokaryotic Genome Annotation Pipeline (PGAP) (12) v4.11 (http://www.ncbi.nlm.nih.gov/genome/annotation_prok/). CBC-LB69 was found to contain a total of 1,591 coding genes and 60 tRNA genes, while CBC-LB8 contained 1,586 coding genes and 61 tRNA genes.

### Data availability.

These whole-genome shotgun projects have been deposited at DDBJ/ENA/GenBank under the accession numbers JABWGR000000000 and JABWOS000000000. The versions described in this paper are the first versions, JABWGR010000000 and JABWOS010000000. The raw files were deposited in the SRA under the accession numbers SRR12037316 and SRR12037315.
